# Safety and Preliminary Efficacy of Lorcaserin for Cocaine Use Disorder: A Phase I Randomized Clinical Trial

**DOI:** 10.3389/fpsyt.2021.666945

**Published:** 2021-07-02

**Authors:** Sade E. Johns, Lori Keyser-Marcus, Antonio Abbate, Edward Boone, Benjamin Van Tassell, Kathryn A. Cunningham, Noelle C. Anastasio, Justin L. Poklis, Tatiana Ramey, F. Gerard Moeller

**Affiliations:** ^1^Institute for Drug and Alcohol Studies, Virginia Commonwealth University, Richmond, VA, United States; ^2^Pauley Heart Center School of Medicine, Virginia Commonwealth University, Richmond, VA, United States; ^3^Department of Statistical Sciences and Operations Research, Virginia Commonwealth University, Richmond, VA, United States; ^4^Department of Pharmacotherapy and Outcomes Science, Virginia Commonwealth University, Richmond, VA, United States; ^5^Department of Pharmacology and Toxicology, Center for Addiction Research, University of Texas Medical Branch, Galveston, TX, United States; ^6^Department of Pharmacology and Toxicology, Virginia Commonwealth University, Richmond, VA, United States; ^7^Division of Therapeutics and Medical Consequences, National Institute on Drug Abuse, National Institutes of Health, Bethesda, MD, United States; ^8^C. Kenneth and Dianne Wright Center for Clinical and Translational Research, Virginia Commonwealth University, Richmond, VA, United States

**Keywords:** cocaine, craving, safety, lorcaserin, visual analog scale, drug choice, serotonin 5-HT_2*c*_ receptor

## Abstract

**Background and Objectives:** Preclinical studies show serotonin (5-HT) 5-HT_2C_ receptor (5-HT_2C_R) agonists reduce cocaine-seeking and cocaine intake. This study examined safety of the 5-HT_2C_R agonist lorcaserin administered with cocaine in participants with cocaine use disorder (CocUD). Secondarily, subjective response to cocaine and choice of cocaine vs. money were examined.

**Methods:** A double-blind, randomized, placebo-controlled trial of 25 inpatient non-treatment seeking participants with CocUD. Participants were randomized to either lorcaserin (*n* = 17) or placebo (*n* = 8). Primary outcome measures included cardiovascular measures and plasma cocaine levels. Secondary measures of subjective response to cocaine were assessed using a visual analog scale (VAS) and cocaine vs. money progressive ratio choice sessions.

**Results:** Thirteen randomized participants were included in the final analysis. No serious or unexpected adverse events were related to lorcaserin. There were no significant interactions between cocaine and lorcaserin on cardiovascular measures, plasma cocaine, or subjective ratings. After multiple comparisons correction, cocaine significantly increased blood pressure, heart rate, and QTc. Lorcaserin significantly decreased VAS ratings of “feel irritable,” “feel hungry,” and “I am craving.” For the cocaine vs. money choice procedure, there was a significant interaction between choice (cocaine vs. money) and lorcaserin. Participants treated with lorcaserin were more likely to choose cocaine.

**Discussion and Conclusions:** This study showed safety of lorcaserin administered with cocaine but lack of efficacy to reduce the reinforcing effects of cocaine.

**Scientific Significance:** This study is the first to show a disconnect between effects of 5-HT_2C_R agonists on craving and cocaine choice in human cocaine users.

## Introduction

Cocaine use disorder (CocUD) is a significant public health problem in the US. In 2015, an estimated 1.81% of the population used cocaine in the past year, an increase from 1.48% in 2011 (1). Among all drug overdose deaths in 2017, 13,942 involved cocaine (2). Although cocaine use has continued to be a public health concern, to date, there are no FDA approved medications for CocUD. Lorcaserin is an FDA-approved 5-HT_2C_R agonist previously marketed for weight reduction (3, 4). Lorcaserin and other selective 5-HT_2C_R agonists (such as R0 60-0175), reduce cocaine self-administration and cocaine-seeking behaviors in rodents (5, 6) and non-human primates (7, 8), although one study reported failure of lorcaserin to alter the choice of cocaine vs. food (9). One prior human study found no effect of lorcaserin on cocaine motivated behavior, but that study only included a 10 mg daily dose of lorcaserin (10). Thus, there is a great need and many opportunities for medication development for CocUD, and preclinical data suggest the possibility that the serotonin 5-HT_2C_ receptor (5-HT_2C_R) agonist lorcaserin may add value in the treatment of CocUD (11–14) but, there is conflicting preclinical and human data on lorcaserin's cocaine effects as a potential abstinence inducing medication.

The overarching aim of the present study was to extend our understanding of the safety of lorcaserin in combination with cocaine and the efficacy of lorcaserin to alter the subjective response to cocaine as well as cocaine self-administration in non-treatment seeking participants with CocUD. The current study was modeled after our previous study for medication safety and efficacy in participants with CocUD (15). The primary outcome measure was the assessment of the interaction between lorcaserin and cocaine in healthy cocaine-using participants, as assessed by changes in their blood pressure, heart rate, and ECG readings, as well as changes in plasma cocaine levels after lorcaserin vs. placebo. Secondary outcomes included the effects of lorcaserin on subjective responses to cocaine, as measured by the VAS during cocaine infusions compared to saline, and choice of IV cocaine vs. money in a cocaine choice procedure.

## Methods

The study was a single center, double-blind, randomized placebo-controlled phase 1b/2a trial including a total of 25 participants who met current Diagnostic and Statistical Manual of Mental Disorders (DSM-5) criteria for CocUD of at least moderate severity and were non-treatment seeking. The study was reviewed and approved by the Virginia Commonwealth University Institutional Review Board and an IND was issued by the FDA. The trial was also registered in clinicaltrials.gov (identifier NCT02537873). All participants provided written informed consent prior to screening for eligibility.

### Participants

Eligible participants were between 18 and 59 years of age currently using cocaine *via* smoking or intravenous (IV) route of administration as determined by self-report and had a positive urine drug screen for the cocaine metabolite benzoylecgonine. To be considered eligible, individuals were also required to have resting heart rate below 90 bpm, blood pressure below 140 mm Hg systolic and 90 mm Hg diastolic, electrocardiogram (ECG) showing normal rhythm and conduction. A DSM-5 diagnosis of psychoactive substance use disorder other than cocaine, opioids, marijuana, or nicotine was considered exclusionary (mild to moderate alcohol use disorder was allowed). None of the participants were physically dependent on alcohol or opioids, as determined by tolerance and withdrawal symptoms. DSM-5 psychiatric disorders other than substance use disorder including but not limited to bipolar disorder, major depressive disorder, attention deficit hyperactivity Disorder, or Schizophrenia, or a neurological disorder requiring ongoing treatment and/or making study participation unsafe were exclusionary, as was any previous medically adverse reaction to cocaine and any clinically significant medical disorder; homicidal or suicidal ideation or a history of suicide attempt within the past 6 months; positive HIV test by self-report or history; positive breath alcohol test or urine drug screen positive for drugs of abuse with the exception of cocaine, cocaine metabolites, opioids, and marijuana; known allergy to lorcaserin; and participation in any investigational drug trial within 90 days prior to baseline. Written informed consent was obtained from all participants.

Upon completion of screening, eligible participants were randomized to Group A (placebo only) or Group B (placebo followed by an ascending dose of lorcaserin). Lorcaserin 10 mg was administered once daily increasing to 10 mg twice daily after 7 days. Intravenous cocaine was administered as an initial single-blind 10 mg dose, followed by double-blind ascending doses of 20 and 40 mg with a 0 mg cocaine (9% normal saline solution) infusion given randomly in the sequence to aid in the blinding of investigators and participants. Randomization to lorcaserin vs. placebo (Group A vs. Group B) was undertaken in a 2:1 ratio.

### Lorcaserin and Placebo Preparation and Administration

Lorcaserin HCL 10 mg tablets (Belviq®, Arena Pharmaceuticals, San Diego, California, United States) were placed in gelatin capsules by the research pharmacist prior to administration. Identical placebo gelatin capsules included dextrose within the capsules. Commercially available sterile normal saline for human use was used as a matched placebo for cocaine. While on the unit, oral medication (Lorcaserin or Placebo) was administered at 8:00 a.m. and 8:00 p.m. Single blind placebo was administered on days 1 and 2 for all participants. Beginning on Study day 3, lorcaserin or placebo was administered in a double-blind fashion. Randomization numbers were obtained from www.randomizer.org in a block of four by the study pharmacist, who maintained the randomization assignment list.

On study Days 3–7, Group A participants received one placebo pill twice daily. Group B received one placebo pill in the morning and one matching lorcaserin 10 mg in the evening, for a total daily dose of 10 mg. On study Days 8–11, participants in Group B had a blinded dose increase to 10 mg of lorcaserin twice daily, for a total daily dose of 20 mg. Group A participants continued to receive only placebo doses. Study days 1, 2, 3, 6, 7, 10, and 11 were all completed as inpatient visits (on the Clinical Research Unit), and both morning and evening doses of lorcaserin/placebo were administered by study personnel. Study days 4, 5, 8, and 9 were completed at outpatient visits, and study medication (Lorcaserin/Placebo) morning doses were administered and observed by study personnel, while the evening dose was provided as a “take home” dose. No medication was provided on study day 12 (the final study safety visit).

### Cocaine Administration and Choice Sessions

Cocaine for IV human use was obtained from a National Institute on Drug Abuse (NIDA) contractor and was packaged in 2 mL vials containing 20 mg per mL. Cocaine was prepared by the research pharmacist according to instructions provided by the NIDA contractor and dispensed according to randomization. Ascending doses of IV cocaine (10, 20, and 40 mg) were prepared by diluting cocaine with 20 mL 0.9% sterile normal saline solution. Each dose of cocaine was administered over 2 min. Doses of lorcaserin and cocaine were not adjusted for body weight. All cocaine administration and choice procedures took place on the Clinical Research Unit of the Wright Center for Clinical and Translational Research at Virginia Commonwealth University.

Intravenous cocaine was administered on day 1 to ensure participant tolerability of the cocaine doses. On study Days 1, 2, 6 and 10, participants were administered ascending doses of IV cocaine, for a total of four IV doses administered on each test day (placebo, 10 mg cocaine, 20 mg cocaine, and 40 mg cocaine), in an escalating dose fashion, with each cocaine administration separated by an hour for safety reasons. The first IV dose (cocaine or placebo) occurred at 9:15 a.m. Baseline placebo cardiovascular measures were collected on day 2. Vital signs, including heart rate, blood pressure, and respiration rate were monitored following placebo/IV cocaine administration. During ascending dose IV cocaine administration, blood pressure, heart rate, ECGs changes, and adverse events were documented as primary outcome measures. The Visual Analog Scale (VAS) was used to assess subjective effects of cocaine. Ratings for each of the following statements on a scale between 0 (not) at all to 100 (extremely), are provided, according to the respondent's present state: “good drug effect,” “bad drug effect,” “feel stimulated,” “feel alert,” “feel energetic,” “feel depressed,” “feel irritable,” “feel clumsy,” “feel sleepy,” “feel tired,” “feel anxious,” “feel restless,” “feel confused,” “feel dizzy,” “feel forgetful,” “feel; hungry,” “feel nauseous,” “have upset stomach,” “have headache,” “have difficulty concentrating,” and “I am craving for cocaine” (16, 17). Participants were administered the VAS at 15 min intervals following cocaine administration during the infusion sessions.

On study Days 3, 7, and 11, subjects participated in two money vs. cocaine choice sessions in lieu of the ascending cocaine dosing procedures described above (18, 19). Each session lasted 4 h and included six choice trials. Choice options were 0 mg of cocaine vs. money ($5.00) for one of the sessions, and 25 mg cocaine vs. money ($5.00) for the other session (participants were blinded to cocaine dose). Session order was determined by random assignment. Cocaine dose of 25 mg was used in the present study as it was previously noted to produce a significant medication effect on cocaine self-administration (18). VAS ratings and vital signs were obtained prior to each session. Participants then completed six choice trials.

The first trial in each session was considered a “sample” during which the participant responded on a keyboard under a fixed ratio (FR) schedule to receive [both money and IV drug (cocaine or saline)]. The remaining five choice trials were spaced 15-min apart. During each trial participants were instructed to choose between self-administering cocaine equivalent to the sample dose (0 or 25 mg) or receiving ($5.00) money. Using a modified progressive ratio scale of responding, the initial response threshold was FR 200 (keyboard presses). Following each choice, the response threshold for the chosen option increased by 400 presses, while the threshold for the non-chosen option remained unchanged. Thus, if a participant only selected cocaine or money, the response requirements escalated progressively through 600, 1,000, 1,400, 1,800, and 2,200 keyboard presses (19).

Infusions were made over a 2-min period followed by a 13-min time-out period. Participants received the money or cocaine dose immediately after meeting the key press threshold for each choice, providing vital signs remained within the predetermined safety range. A maximum of 150 mg of IV cocaine or $30 could be obtained in a given session.

Cocaine pharmacokinetics samples were obtained on study Days 2, 6, and 10, during single -blind placebo administration over the course of 6 h after the cocaine infusion. Plasma concentration-time profiles of cocaine infusion were analyzed to obtain pharmacokinetic parameter estimates of cocaine following doses of placebo (day 2), 10 mg lorcaserin (day 6), and 20 mg of lorcaserin (day 10). The method for analysis is described elsewhere (20). See Table 1 for procedures by study day.

**Table 1:** Study procedures.

**Study day(s)**	**Study drug dosing^ʈ^**	**Morning procedures**	**Afternoon procedures**
Study Day 1 (Inpatient)	Placebo (8 a.m.)	- Three screening doses of cocaine (testing for tolerability)	None
Study Day 2 (Inpatient)	Placebo (8 a.m.) Placebo (8 p.m.)	- 10 mg cocaine for PK measures	- Three doses of cocaine - Baseline cardiovascular measures - Subjective ratings (VAS)
Study Day 3 (Inpatient)	Placebo (8 a.m.) Lorcaserin 10 mg OR Placebo (8 p.m.)	- VAS - Cocaine self-administration session (baseline)	- VAS - Cocaine self-administration session (baseline)
^*^Study Days 4 and 5 (Outpatient)	Placebo (8 a.m.), Lorcaserin (8 p.m.) OR Placebo (8 a.m.), Placebo (8 p.m.)	- Outpatient medication and check-in visit	None
Study Day 6 (Inpatient)	Placebo (8 a.m.), Lorcaserin (8 p.m.) OR Placebo (8 a.m.), Placebo (8 p.m.)	- 10 mg Cocaine for PK, lorcaserin blood levels	- Three Doses of Cocaine - Cardiovascular measures - Subjective Ratings (VAS)
Study Day 7 (Inpatient)	Placebo (8 a.m.), Lorcaserin (8 p.m.) OR Placebo (8 a.m.), Placebo (8 p.m.)	- Cocaine self-administration session	- Cocaine self-administration session - Impulsivity Testing
^*^Study Days 8 and 9 (Outpatient)	Lorcaserin (8 a.m.), Lorcaserin (8 p.m.) OR Placebo (8 a.m.), Placebo (8 p.m.)	- Outpatient medication and check-in visit,	None
Study Day 10 (Inpatient)	Lorcaserin (8 a.m.), Lorcaserin (8 p.m.) OR Placebo (8 a.m.), Placebo (8 p.m.)	- 10 mg cocaine for PK, lorcaserin blood levels	- Three doses of cocaine - Cardiovascular measures - Subjective Ratings (VAS) - Impulsivity Testing
Study Day 11 (Inpatient)	Lorcaserin (8 a.m.), Lorcaserin (8 p.m.) OR Placebo (8 a.m.), Placebo (8 p.m.)	- Cocaine self-administration session	- Cocaine Self Administration
Study Day 12 (Inpatient)	None	- Impulsivity Testing	Physical exam
Study Days 15–19 (Outpatient)	None	- Physical exam (Day 19 only)	

*All study visits (inpatient and outpatient) included vitals, UDS, and ECG. Inpatient visits also included physical exam and continuous cardiovascular monitoring*.

ʈ *Medication on study days 3–11 based on study drug randomization assignment (Lorcaserin or placebo)*.

* *On these days the AM medication dose taken at visit, and PM dose provided with instructions to take at home at 8 p.m*.

### Statistical Analyses

All analyses were completed using SPSS version 27 and R. Baseline differences between loracaserin and placebo groups were made using *t*-tests and chi-square calculations. Subjective responses on the VAS, blood pressure, heart rate, and QTc after doses of cocaine when administered lorcaserin vs. placebo were examined using a generalized linear mixed model analysis with AR1 covariance structure, with the participant as a random effect for each outcome and a Kenward Roger approximation. Potential interactions between lorcaserin dose and cocaine choice, were explored using a generalized linear model analysis, with number of cocaine choices as the dependent variable with drug available during choice session (cocaine vs. saline) and lorcaserin dose (0, 10, and 20 mg) as the independent variables. Main effects and interactions between drug available and lorcaserin dose were also explored. A repeated measures ANOVA was performed on the natural logarithm transformed variable for cocaine blood plasma level with a random effect for participant and time since administration treated as a block factor to examine the influence of lorcaserin on blood plasma cocaine levels.

Primary outcome measures (diastolic blood pressure, systolic blood pressure, heart rate, QTc, and plasma cocaine levels) were corrected for multiple comparison using a Bonferroni test, with 4 cardiovascular variables and 1 plasma cocaine variable, resulting in a comparison wise error rate of 0.01. The VAS secondary outcome measure produced 21 variables of interest, necessitating a Bonferroni correction rate of 0.002.

## Results

**Figure 1:**
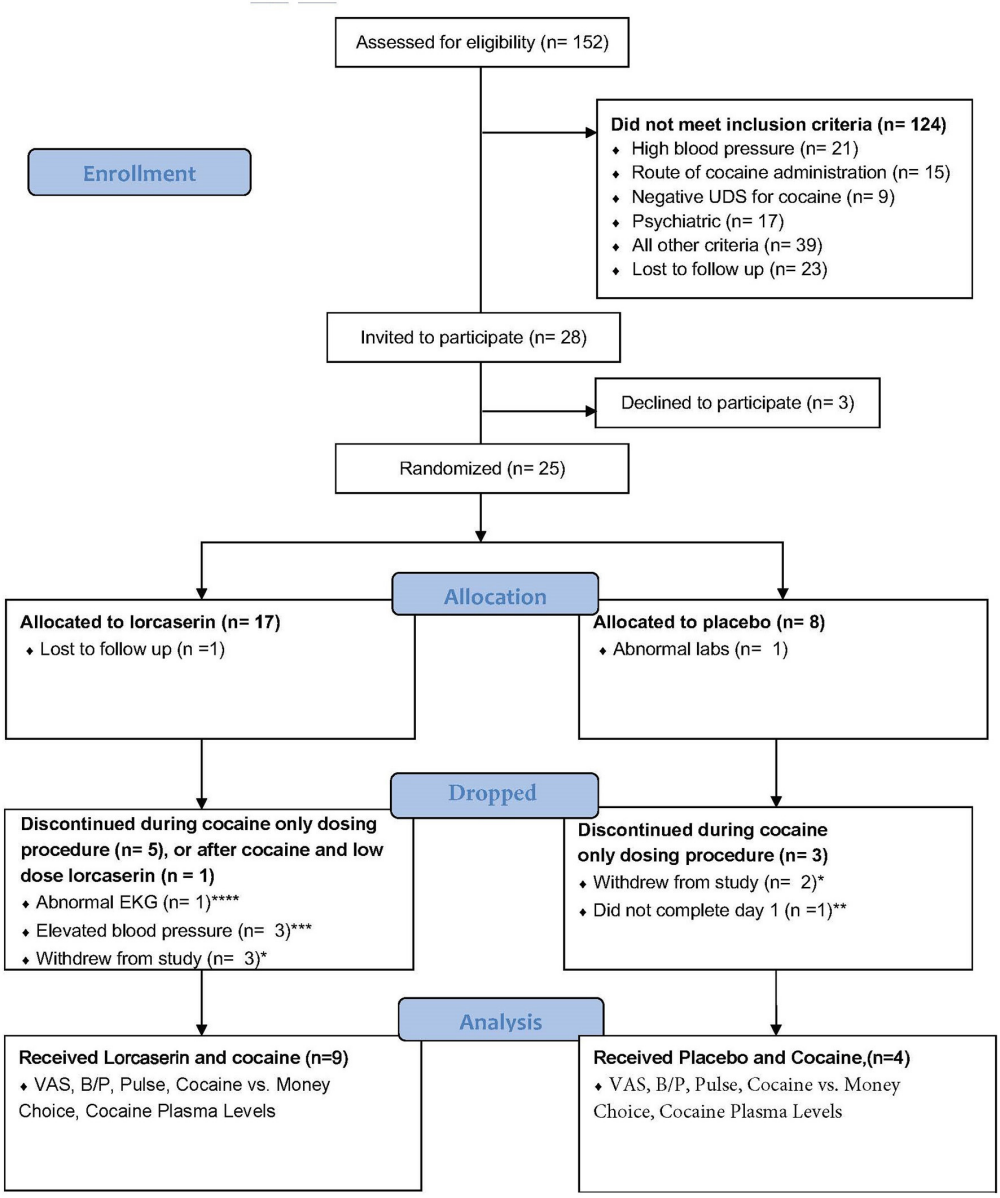
Flowchart according to the Consolidating Standard of Reporting Trials (CONSORT) reporting completion of study and subject inclusion. *Five subjects voluntarily withdrew from the study. **One subject experienced paranoia after cocaine dose. ***Occurred after the baseline dose of cocaine but before lorcaserin administration. ****Rate dependent QTc prolongation.

Participant enrollment and completion details are shown in Figure 1. Twenty-five participants were randomized to treatment with either lorcaserin (*n* = 17) or placebo (*n* = 8). The intervention was discontinued in seven of the participants randomized to lorcaserin and three of the participants randomized to placebo for reasons unrelated to lorcaserin (see Figure 1). Thirteen of the randomized participants completed all the procedures of the study, including nine participants for the lorcaserin group (eight males and one female, mean age = 47.8 ± 5.29) and four participants for the placebo group (three males and one female, mean age = 56.3 ± 2.06). Two participants were dropped post-randomization but prior to receiving medication, due to no-show/repeated unsuccessful attempts to contact (*n* = 1), and abnormal ECG (*n* = 1). Five participants voluntarily withdrew from the study before study day 5 completion, and one participant was dropped on study day 1 due to experiencing paranoia after the baseline cocaine dosage (prior to lorcaserin administration). As such, their data were excluded from study analyses.

Descriptive statistics were run for the sample and are presented in Table 2. No between-group differences were noted for number of years of cocaine use, alcohol use, cigarette, or marijuana use. Age was significantly different, with the placebo group being older than the lorcaserin group. There was no significant interaction between lorcaserin and cocaine on cardiovascular parameters.

**Table 2:** Demographics.

	**Lorcaserin group (*n* = 9)**	**Placebo group (*n* = 4)**	***p***
Gender (males/females)	8/1	3/1	0.522
Age, mean ± SD	47.8 ± 5.29	56.3 ± 2.06	0.011^*^
Race (African American/Caucasian)	7/2	4/0	0.285
Weight (lbs.) mean ± SD	62 ± 6.8	171 ± 34.1	0.639
Number of regular cocaine users	9	4	
Years of cocaine use, mean ± SD	15.7 ± 10.2	20.75 ± 10.2	0.429
Number of regular alcohol users	4	1	
Years of alcohol use, mean ± SD	15.8 ± 14.7	9.50 ± 17.1	0.512
Number of regular cigarette smokers	4	2	
Years of smoking use, mean ± SD	20.4 ± 15.0	32.0 ± 11.8	0.203
Number of regular marijuana smokers	6	2	
Years of marijuana use, mean ± SD	17.6 ± 14.4	18.0 ± 9.17	0.962

*SD, standard deviation*;

* *p ≤ 0.05*.

**Figure 2:**
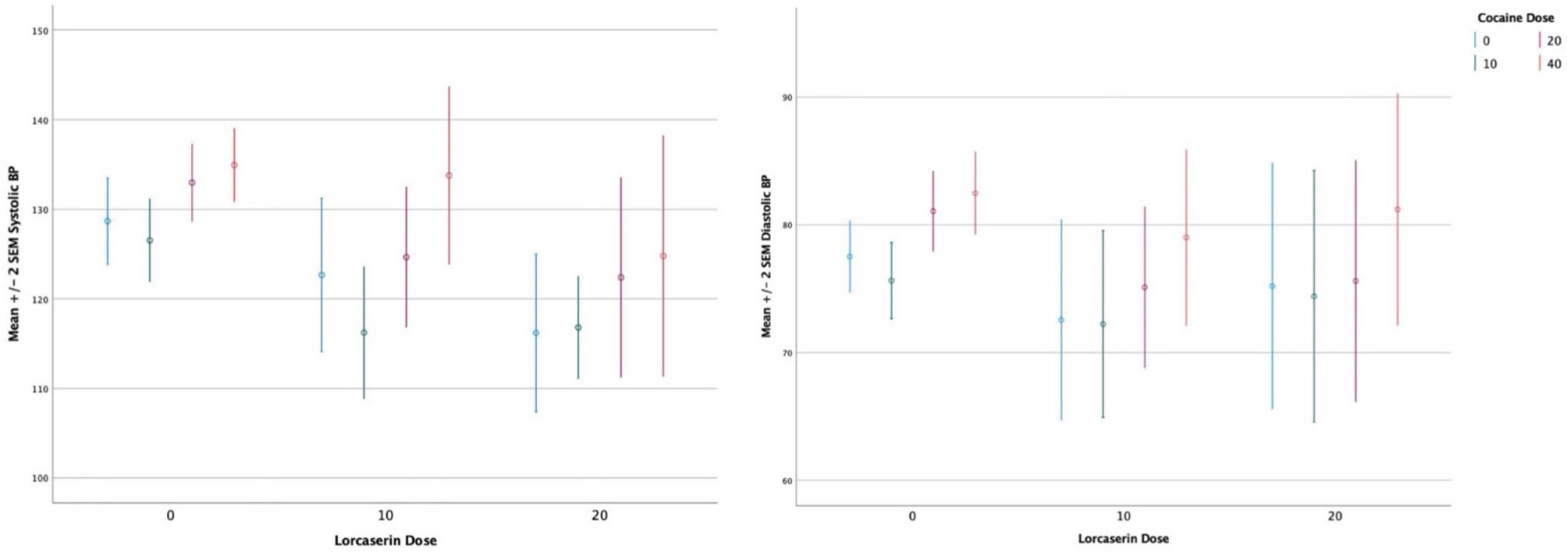
Effect of cocaine and lorcaserin dose on blood pressure.

A main effect of cocaine dose on systolic blood pressure (F = 11.2, df = 3, *p* < 0.0010), diastolic blood pressure (F = 8.5, df = 3, *p* < 0.000) (see Figure 2), heart rate (F = 18.4, df = 3, *p* < 0.0010), and QTc (F = 4.17, df = 3, *p* < 0.0080) persisted after correction for multiple comparisons, with cocaine increasing all measures. There was no significant main effect of lorcaserin dose on systolic blood pressure (F = 1.00, df = 3, *p* = 0.373), diastolic blood pressure (F = 0.720, df = 3, *p* = 0.492) heart rate (F = 1.86, df = 3, *p* = 0.167), and QTc (F = 2.07, df = 3, *p* = 0.136). No sustained changes in rhythm or in ST segment in lorcaserin or placebo treated groups were noted. One participant randomized to lorcaserin had a >30 ms increase in QTc during the cocaine choice procedure. This occurred at baseline (prior to initiating lorcaserin) and after treatment with 10 mg daily of lorcaserin. The change in QTc was did not differ between pre/post lorcaserin administration and was considered unrelated to lorcaserin, but instead related to a rate dependent increase in QTc.

For the interaction between lorcaserin and cocaine on plasma cocaine levels, a likelihood ratio test revealed that the cocaine by lorcaserin interaction was not statistically significant (χ^2^>8.53, *df* = 8;*p* = 0.384). None of the interactions between cocaine and lorcaserin were statistically significant at the 0.05 significance level for any of the endpoints on the VAS collected during the IV cocaine administration sessions. Based on this finding, the main effects can be directly assessed.

Three VAS ratings survived correcting for multiple comparisons: a decrease in “feel irritable” (*p*, 0.0020), “feel hungry” (*p*, 0.0001), and “I am craving” (*p*, 0.0001) for lorcaserin. No VAS ratings survived correction for multiple comparisons for cocaine. A significant interaction between choice (cocaine vs. money) and lorcaserin dose (*p* = 0.019) was noted for the cocaine choice tasks, with participants receiving lorcaserin being more likely to choose cocaine. As seen in Table 3, participants opted for cocaine over money an average of 1.71 choices (out of 5) per session at 0 mg lorcaserin. However, at both 10 and 20 mg lorcaserin, the average choice of cocaine increased to 4.29 choices/session.

**Table 3:** Mean drug choices per session by lorcaserin dose.

**IV drug administered**	**Lorcaserin dose (mg)**	**Mean**	**Std Error**	**95% CI**	
				**Lower**	**Upper**
Saline	0	0.670	0.334	0.010	1.32
	10	0.750	0.541	−0.310	1.81
	20	1.86	0.578	0.720	2.99
Cocaine	0	1.71	0.334	1.06	2.37
	10	4.29	0.578	3.15	5.42
	20	4.29	0.578	3.15	5.42

## Discussion

The present study provided support for the potential safety of lorcaserin use among individuals who use cocaine, as no difference in number or severity of adverse events were noted when lorcaserin was given in conjunction with cocaine. The current results also revealed reductions in cocaine craving, hunger, and irritability subjective ratings associated with lorcaserin dose. However, lorcaserin increased the choice of cocaine in a cocaine vs. money choice procedure.

The current study shares some findings with another recent double-blind study in nine male participants with CocUD, in which lorcaserin decreased craving after IV cocaine (10). However, the Pirtle et al., study results differed in that lorcaserin 10 mg did not alter cocaine or monetary reinforcement but did increase the overall task response latency and latency for the first choice of cocaine but not money (10). Possible reasons for differences between our results include our use of repeated administration of lorcaserin over several days increasing to 10 mg twice daily vs. single dose administration in the Pirtle et al., study. In addition, the two studies differed regarding the choice procedure paradigm such that subjects had to “work harder” in our study to receive either a dose of cocaine or $5.00 on a progressive ratio schedule.

In as much as preclinical findings of cue induced responding may be translational to human craving, the currently evidenced reduction in craving produced by lorcaserin is consistent with preclinical studies showing a decrease in cocaine cue induced responding in rodents and non-human primates treated with 5-HT_2C_R agonists (5–8). However, the present results are at odds with earlier studies demonstrating reduced cocaine self-administration following lorcaserin. A recent publication reviewing the lack of translation for lorcaserin from preclinical to clinical studies, and recommending drug choice procedures as one method to improve translation is noteworthy and provides a rationale for this discrepancy (21). Further, a preclinical study by the same authors using the choice of cocaine vs. food found that repeated lorcaserin did not significantly alter percent cocaine choice (9).

This phenomenon is not new. The disparity between craving and drug taking has been observed with other pharmacotherapies. Most notably, acamprosate has been shown to reduce craving for alcohol, but not reduce alcohol use in active drinkers (22, 23), leading to its use for relapse prevention rather than abstinence initiation. It is possible that 5-HT_2C_R agonists may be better suited to relapse prevention for cocaine use disorder. To lend support to this theory, multiple preclinical studies have demonstrated 5-HT_2C_R agonists reduce impulsivity (14, 24, 25). We have previously shown that impulsivity is a predictor of treatment outcome in cocaine use disorder participants (26). Reductions in impulsivity may also contribute to relapse prevention. To date, few studies have examined any pharmacotherapy to support relapse prevention among individuals with cocaine use disorder who are in early remission. Further research in this area is warranted.

A few limitations of the present study must be acknowledged. First, the small sample size inhibits drawing firm conclusions regarding lorcaserin's impact on the arrhythmic risk of cocaine. However lack of cardiovascular effects of lorcaserin in the present study is consistent with results of the large Phase 3 clinical trial of lorcaserin in 12,000 high-risk obese patients with established cardiovascular disease (27). Another limitation is the relative lack of female participants (*n* = 2), which prohibits examining the potential role of sex on outcomes. Further, the potential role of route of cocaine administration warrants discussion. Participants in the present study reported smoking (crack) cocaine (rather than IV cocaine use, as was used in the study design). Prior research provided a rationale for including both smoked cocaine users and IV cocaine users in study samples, based upon the similar rapid onset of action for both forms of administration and lack of effect of IV cocaine administration on subsequent cocaine use in smoked cocaine users [described in detail elsewhere (28)]. It must also be noted that due to safety concerns, the order of lorcaserin dose testing was ascending. Finally, the choice sessions occurred on different days than IV cocaine ascending dose sessions, in which VAS ratings were measured.

## Conclusion

This study exploring the safety and early phase efficacy of lorcaserin in participants with CUD provides mixed evidence, with a reduced subjective craving but an increased choice for cocaine, without cardiovascular interactions of the cocaine-lorcaserin combination. A recent FDA drug safety communication about the increased risk of cancer with lorcaserin led to the voluntary withdrawal of the medication from the US market (29). Hence, lorcaserin will not be available for use in treatment of patients with CocUD. As a representative of 5-HT_2C_R agonists class, these data do not support further study on the potential role of that class of compounds as an abstinence initiation medication. However, the potential role of 5-HT_2c_R agonists in supporting continued abstinence among individuals with CocUD merits further exploration.

## Data Availability Statement

The de-identified raw data supporting the conclusions of this article will be made available by the authors, without undue reservation.

## Ethics Statement

This study involving human participants was reviewed and approved by Virginia Commonwealth University Institutional Review Board (VCU IRB). Study participants provided written informed consent to participate in this study.

## Author Contributions

EB organized the database. EB and FGM performed the statistical analyses. SJ wrote the first draft of the manuscript. All authors contributed to the conception and design of the study, manuscript revision, and read and approved the submitted version.

## Conflict of Interest

NA and KC have current funding from VidaLibreBio, Inc., for research unrelated to this study. The remaining authors declare that the research was conducted in the absence of any commercial or financial relationships that could be construed as a potential conflict of interest.
